# The Lunacy Bill

**Published:** 1899-05-06

**Authors:** 


### The Lunacy Bill.

The Bill which has been introduced into the House of Lords by the Lord Chancellor for the amendment of the Lunacy Laws, and which has passed the second reading in that assembly, represents the third attempt of the Government to effect necessary reforms in this department of legislation. On two former occasions a Bill much resembling the present one has been introduced in the same manner, has passed the third reading, and has been excluded by the press of other (we will not say more important) business from receiving consideration in the House of Commons. The noble and learned lord who is responsible for the present measure is not believed to be very much in earnest about it, or even to be very intimately acquainted with its provisions, for he is reported to have told the House of Lords that these were exactly the same as those which had been accepted in two previous sessions. In the main, this is the case; but the new Bill contains one provision which, although it has long been in force in Scotland, is wholly new to the law of this country, and which, moreover, is contained in clauses so short and unobtrusive that they might easily escape the observation of any but a critical observer. This provision is that a patient who is threatened with insanity, but not actually insane, may be certified by a medical practitioner, not as a lunatic, but as a person who, without proper treatment, is likely to become one; and may be committed, for a period not exceeding six months, to the care of a person to be named on the certificate. The idea is to afford facilities for the timely treatment of incipient or threatened lunacy, without affixing any corresponding stigma upon the patient, and without the present necessity of waiting until the symptoms are confirmed, and the time for efficacious intervention has to some extent passed away. No details of the proposed arrangements are given in the Bill, which merely provides that, under the certificate described, and while it remains in force, certain specified clauses of existing Acts are to apply. It is hardly worth while to discuss in detail a clause which, even if the Bill containing it should reach the House of Commons, would almost certainly receive some modification in Committee; but the experience of Scotland is entirely favourable to the adoption of the principle involved, which may be regarded as a distinct advance on English legislation, although probably calling for some better-defined safeguards than the Bill in its present form appears to contain.

Another proposal, to which great exception will be taken by all who are concerned in the treatment of the insane, is that the period of seven days which may now elapse between the signing of an urgency order for the reception of a lunatic and the completion of all the formalities necessary for his permanent detention shall be reduced to four days, and to four days from the date of signature of the urgency order; so that, if the latter for any reason could not be acted upon until the day after it was dated, the period for completion would be reduced to three days from that of the actual reception of the patient. The time now allowed is found in practice to be too short rather than too long, partly because the permanent order cannot be signed by all magistrates, but only by those who have been appointed for the purpose, and who are not always accessible at the time when they are wanted, or who, on account of various scruples, are apt to throw difficulties in the way of completion which time is necessary to remove. It is feared that, if this change were to become law, many dangerous lunatics would be released after four days' confinement under an urgency order, with morbid feelings or hatreds intensified by their brief incarceration; and it is pointed out that in Scotland, where the time afforded for completion is only three days, the order can be signed by any sheriff or sheriff's deputy, and that one or other of these officials is always to be found when wanted. It is thought that the difficulty anticipated in England might be lessened, if not overcome, if power to sign an order were conferred upon every magistrate, instead of being, as at present, restricted to a few.

